# Benefits of red blood cell transfusion in patients with traumatic brain injury

**DOI:** 10.1186/s13054-019-2498-2

**Published:** 2019-06-13

**Authors:** Weimin Zhang, Kailei Du, Xuping Chen

**Affiliations:** Intensive Care Unit, Dongyang People’s Hospital, NO.60 Wuning West Road, Jinhua City, Zhejiang 322100 People’s Republic of China

To the editor,

Anemia is a common clinical condition in patients suffering from traumatic brain injury (TBI). As low hemoglobin level may increase the risk of poor brain oxygen delivery and secondary ischemic injury in TBI, red blood cell (RBC) transfusion is often applied in postoperative intensive care. However, the benefits remain debated. Dozens of cohort studies were performed to investigate the association between RBC transfusion and clinical outcomes, such as mortality and long-term neurological function. However, the conclusions were conflicting which is largely due to the great heterogeneity. For instance, the hemoglobin targets vary greatly, from 6 to 12 g/dl, using one hemoglobin value to represent the whole hemoglobin level; there is no specific RBC transfusion protocol and unadjusted confounding factors. Of course, aiming to address these limitations, randomized controlled trials (RCT) were also conducted. Aiming to provide a systematic review, we conducted a literature search on PubMed and Embase, without limitations. Only three RCTs were identified investigating the RBC transfusion efficacy in patients with TBI, and several limitations should be noticed. First is the mortality rate. All the three RCTs reported the mortality rate, and McIntyre et al. [1] and Robertson et al. [2] found no significant difference in overall mortality, while in Gobatto et al.’s [3] study, 44 TBI patients were included and a significant reduction of mortality was found (7/23 vs. 1/21, *p* = 0.048). In the meta-analysis, the pooled outcome also showed a non-significant conclusion, with significant heterogeneity. Noteworthy, we noticed all the death in Gobatto et al.’s study occurred during ICU stay. However, in clinical practice, a significant proportion of TBI patients may die shortly after admission because of severe brain damage which may also explain the fact that in the other two RCTs, 60% [1] of death occurred during ICU stay and more than 60% of death [2] occurred within 13 days after admission (Fig. 3 in Robertson et al.’s [2] study). Furthermore, despite 7 g/L and 9 g/L were defined as the restrictive and liberal transfusion targets, the hemoglobin levels are almost the same within the first 4 days in Gobatto et al.’s study (Fig. 2). Thus, the timing of death of these patients should be presented as the inclusion of these patients may lead to a biased conclusion. Second, the GOS was commonly used as an index for long-term neurological outcome (Fig. 1). In our meta-analysis, no significant improvement was found both in subgroups including and excluding death. Based on the current evidence, the debate of RBC transfusion remains unsettled. Well-designed multicenter investigations are needed to reach a stable conclusion.

**Fig. 1:**
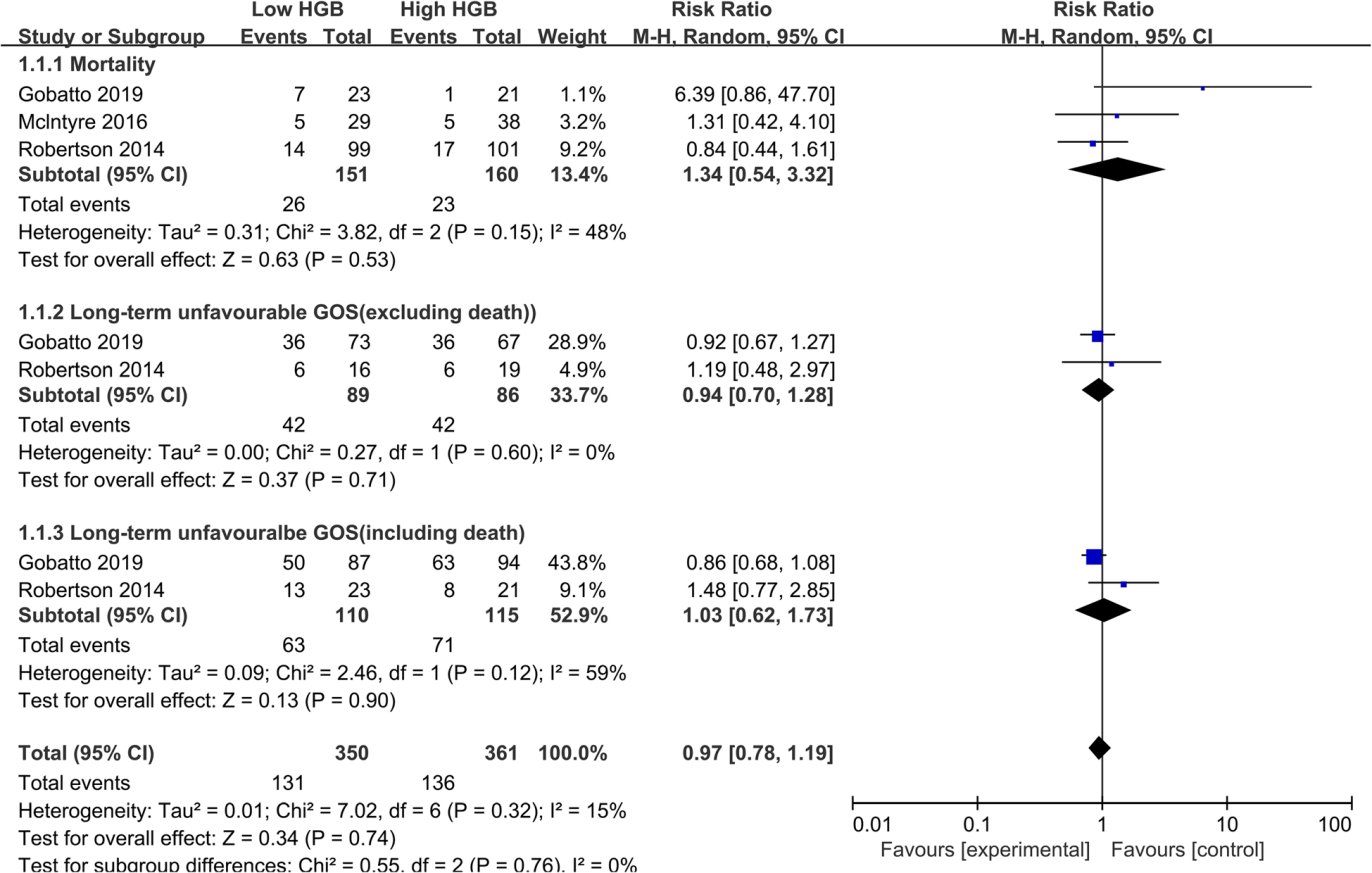
Forest plot of subgroup comparisons of mortality and GOS. GOS Glasgow Outcome Scale

## Data Availability

Not applicable.
