# Supplementary material for: Benefits of red blood cell transfusion in patients with traumatic brain injury
Source: Crit Care. 2019 Jun 13;23:218. doi: 10.1186/s13054-019-2498-2 (PMC6567573; doi:10.1186/s13054-019-2498-2)
Supplement: Related texts

# Reply: Authors’ response Red blood cells’ transfusions and mortality in traumatic brain-injured patients

**Authors:** André L. N. Gobatto, Milena A. Link, Davi J. F. Solla, Luiz M. S. Malbouisson

To the editor,

The letter from Dr. Zhang et al. is very interesting and gives us the opportunity to explore some details regarding the relationship between transfusion and mortality in patients with traumatic brain injury (TBI).

To date, only three randomized clinical trials (RCTs) have evaluated blood transfusion strategies in patients with TBI, with different results. The trials by McIntyre et al. [1] and Robertson et al. [2] found no significant difference in overall mortality, while in our study [3] a significant reduction of mortality was found (7/23 vs. 1/21, *p* = 0.048). Part of these results might be explained by different design, inclusion criteria, and patient populations. The trial by McIntyre et al. [1] is actually a sub analysis of the TRICC trial, evaluating 67 TBI patients from the main 838 patients cohort, randomized to a liberal (Hb > 7.0 g/dL) or conservative (Hb > 10 g/dL) transfusion strategy. The trial included stable, resuscitated patients in the intensive care unit (ICU) and was not designed to study TBI patients. Moreover, in the trial by Robertson et al. [2], anemia was not an inclusion criterion and the patients in both groups had average hemoglobin concentrations (Hb) greater than 9 g/dL at all reported time points (e.g., Hb 9.7 vs. 11.4 g/dL at day 9, in restrictive and liberal groups, respectively), which may have precluded adequate assessment of the effects of the restrictive transfusion strategy.

By contrast, in TRAHT, by including only TBI patients with a hemoglobin concentration under 9 g/dL, we created a difference between the groups, using a real restrictive transfusion strategy in the control group. Most TBI patients are not severely anemic at ICU admission and develop anemia during ICU stay. In our trial, the mean hemoglobin concentration during the first 14 days after hospital admission was 9.3 ± 1.3 g/dL in the liberal group and 8.4 ± 1.0 g/dL in the restrictive group (*p* < 0.01), giving a mean difference of 0.9 ± 0.2 g/dL. This difference gradually increased after the fourth day, to a peak on the tenth day, when the difference was 1.8 ± 0.4 g/dL (CI 95% 1.0–2.6, *p* < 0.01). The mean Hb at ICU admission was 10.2 ± 1.4, similar between groups, as well as during the first 3 days. The median time from ICU admission to randomization was 3 [2–4] days, which is compatible with the difference in Hb levels between groups being clearer from the 4th day on.

In our trial, most deaths occurred during ICU stay (7/23 vs. 1/21, *p* = 0.048). At 6-month follow-up, one patient from the liberal group died (7/23 vs. 2/21). In a post hoc analysis, the hospital deaths occurred at a median of 9 [7.5–14.5] days, a similar time point as compared to the Robertson’s study. At the 6-month follow-up, the restrictive group mean survival was 117.7 ± 18.7 vs. 169.2 ± 9.6 days for the liberal group (log-rank test, *χ*² = 3.223, *p* = 0.073) (Fig. 2).

**Fig. 2:** Survival analysis for the 180-month follow-up

TRAHT was a pilot trial, aimed at evaluating the feasibility of a randomized clinical trial comparing liberal and restrictive blood transfusion strategies in patients with moderate and severe TBI. Although its secondary analysis in favor of the liberal transfusion strategy were noteworthy, we agree the study results should be interpreted cautiously and well-designed multicenter RCT are still necessary.
